# Subliminal action priming modulates the perceived intensity of sensory action consequences^☆^

**DOI:** 10.1016/j.cognition.2013.11.008

**Published:** 2014-02

**Authors:** Max-Philipp Stenner, Markus Bauer, Nura Sidarus, Hans-Jochen Heinze, Patrick Haggard, Raymond J. Dolan

**Affiliations:** a Wellcome Trust Centre for Neuroimaging, University College London, London WC1N 3BG, UK; b Department of Neurology, University of Magdeburg, 39120 Magdeburg, Germany; c Institute of Cognitive Neuroscience, University College London, London WC1N 3AR, UK

**Keywords:** NCE, negative compatibility effect, PCE, positive compatibility effect, Sensory attenuation, Sense of agency, Motor predictions, Subliminal motor priming, Negative compatibility effect

## Abstract

The sense of control over the consequences of one’s actions depends on predictions about these consequences. According to an influential computational model, consistency between predicted and observed action consequences attenuates perceived stimulus intensity, which might provide a marker of agentic control. An important assumption of this model is that these predictions are generated *within the motor system*. However, previous studies of sensory attenuation have typically confounded motor-specific perceptual modulation with perceptual effects of stimulus predictability that are not specific to motor action. As a result, these studies cannot unambiguously attribute sensory attenuation to a motor locus. We present a psychophysical experiment on auditory attenuation that avoids this pitfall. Subliminal masked priming of motor actions with compatible prime–target pairs has previously been shown to modulate both reaction times and the explicit feeling of control over action consequences. Here, we demonstrate reduced perceived loudness of tones caused by compatibly primed actions. Importantly, this modulation results from a manipulation of motor processing and is not confounded by stimulus predictability. We discuss our results with respect to theoretical models of the mechanisms underlying sensory attenuation and subliminal motor priming.

## Highlights

• In theory, the motor system attenuates action-outcome perception to signal agency.
• We use subliminal motor priming to test for the predicted motor locus of this effect.
• The perceived intensity of an action-outcome is attenuated by compatible priming.
• Compatible priming is known to enhance explicit agency judgements.
• Sensory attenuation and agency inference depend on overlapping motoric signals.

## 1 Introduction

“Sense of agency” refers to the pre-reflective feeling of controlling one’s actions and, through them, sensory events in the outside world (Haggard & Chambon, 2012). Sense of agency is a fundamental aspect of human action control, as illustrated by the profound changes when actual control and subjective experience of agency are decoupled, as in schizophrenia (Voss et al., 2010) or in patients with alien motor phenomena (Spence, 2002).

According to an influential theoretical account of the sense of agency, agency is inferred by comparing predictions made during action processing with perception (e.g. Shergill, Samson, Bays, Frith, & Wolpert, 2005; but see Synofzik, Vosgerau, & Newen 2008). Based on computational theories of motor control (Wolpert & Miall, 1996), such comparator models assert that a sense of agency over an external event will occur when the sensory information about the event is a predicted consequence of the action. According to the model, predictions of action consequences are used to cancel or partly cancel sensory feedback signals at a comparator node. This cancellation or attenuation process reduces perceived stimulus intensity of only those external events that are caused by one’s own actions (e.g. Blakemore, Wolpert, & Frith, 1998; but see Brown, Adams, Parees, Edwards, & Friston, 2013). When a sensory event is not predicted by current action processing, there is said to be a ‘mismatch’ between prediction and sensory consequence, and the sensory event is not attenuated. Such a mismatch may signal non-agency (Farrer et al., 2008).

While the comparator model cannot account for all aspects of the sense of agency (Haggard and Chambon, 2012, Synofzik et al., 2008), it provides physiologically plausible and testable predictions of how an integration of motor and sensory processing may contribute to the sense of agency. Sensory attenuation of action consequences is perhaps the most distinctive prediction of this model, and, accordingly, has received considerable attention in the literature (e.g. Bays et al., 2005, Cardoso-Leite et al., 2010). The model also makes the specific prediction that sensory predictions originate from a *motor locus*, within the circuits that generate motor actions. For example, sensory predictions of action consequences were linked to preparatory motor processing in theoretical work by Wolpert and Flanagan (2001) and by Wolpert and Ghahramani (2000). Because these predictions are generated by an internal forward model within the motor system, they are thought to be physiologically distinct from purely associative sensory predictions as induced, for example, by pairing of external, sensory events.

Previous studies have not provided truly convincing evidence for this motor-specificity of sensory attenuation, in our view, although many have claimed to do so. Sensory attenuation effects have been demonstrated for self-applied tactile (e.g. Bays et al., 2005, Blakemore et al., 1999), auditory and visual stimulation (Cardoso-Leite et al., 2010, Weiss et al., 2011). However, these previous studies cannot unambiguously attribute these perceptual phenomena to motor processing. To do this, the physical parameters of the relation between action and consequent stimulus should be identical in the agency and non-agency condition. In particular, any differences between conditions in the contingency between action and stimulus easily would lead to stimulus identity or timing being more predictable in one condition than another. Any such difference in predictability would open the door to non-specific explanations of sensory attenuation phenomena, undermining a motor-specific account.

For example, most previous studies on sensory attenuation have manipulated the sense of agency by comparing conditions which differed regarding (a) the presence of a motor response before the stimulus (e.g. Weiss et al., 2011), (b) its temporal relation to the stimulus (e.g. Bäss, Jacobsen, & Schröger, 2008), or (c) the mapping between different actions and consequent stimuli (e.g. Cardoso-Leite et al., 2010). In many cases, these manipulations of the relation between action and a subsequent sensory event clearly change stimulus predictability. In addition, some contrasts introduce other, potentially more dramatic differences between conditions, such as whether a motor task is present or not or whether contingencies have been learnt. Due to these potential confounds, any sensory attenuation in such studies could reflect purely associative sensory predictions that are not specific to motor action in addition to, or instead of, predictive signals generated within the motor system (for a review, see Hughes, Desantis, & Waszak, 2013).

A recent series of studies has shown that an experimental manipulation of action processing, rather than the physical parameters of the action-stimulus sequence, can influence measures of experienced control over stimuli (Chambon and Haggard, 2012, Wenke et al., 2010). Critically, these studies manipulated experienced agency not by omitting an action or changing the relation between an action and a consequent stimulus, but by inserting masked primes prior to the action. Wenke et al. (2010) and Chambon and Haggard 2012 demonstrated that self-reports of control over a visual stimulus that is contingent on an action decreases when subliminal primes and targets are incompatible when compared to trials with compatible prime–target pairs. These studies remove many of the potential confounds mentioned above. In particular, an action is always present, and its physical relation with the consequent event is held constant. Therefore, non-specific explanations based on stimulus predictability cannot readily explain the result. However, both of these studies focused on explicit agency judgements, i.e., self-reports of experienced causal control over a stimulus. In contrast, sensory attenuation is a pre-reflective and implicit measure, which may provide more direct insight into sense of agency (Synofzik et al., 2008).

Here, we combined subliminal masked priming of motor actions with a psychophysical measure of sensory attenuation. Our motivation for this study was threefold: Firstly, we wanted to develop a theoretically pure, well-controlled test of sensory attenuation that avoided confounding effects of stimulus predictability. Secondly, we wanted to test the theoretical prediction that sensory attenuation arises specifically from motor processing. And thirdly, we wanted to test whether differences in action processing can lead to differences in sensory attenuation, even when the objective contingency of a stimulus on the preceding action is held constant. Such results would provide more convincing evidence for sensory attenuation as a motor-specific aspect of sense of agency.

Prime–target compatibility in subliminal priming tasks can have either facilitatory or inhibitory effects on reaction times and error rates, depending on the stimulus onset asynchrony (SOA) of primes and targets (Eimer, 1999; Schlaghecken & Eimer, 2000). At longer SOAs, facilitatory effects of compatible primes (positive compatibility effects, PCE) turn into inhibitory effects (negative compatibility effects, NCE). Chambon and Haggard (2012) reported similar effects of prime–target compatibility on control ratings for both PCE and NCE conditions, i.e., the experience of control was diminished by incompatible primes irrespective of the prime–target SOA. In particular, primes at NCE latency had a positive, enhancing effect on the sense of control over action outcomes, despite having a negative, detrimental effect on reaction times. This result suggests that sense of agency may depend on how the prime biases the initial process of action selection, and not on how the prime influences actual subsequent execution.

Here, we focus on priming conditions known to induce negative compatibility effects on motor performance. This choice was motivated by the strong overlap between the neural mechanisms underlying the negative compatibility effect and those underlying sensory attenuation. Both effects have been linked to the supplementary motor area by correlational and causal evidence (Boy et al., 2010, Boy et al., 2010, Haggard and Whitford, 2004, Sumner et al., 2007, Voss et al., 2008). Positive compatibility effects on motor performance, on the other hand, have been shown to involve more widespread cortical networks, including more lateral motor and attentional areas (D’Ostilio and Garraux, 2012, Dehaene et al., 1998). Thus, the NCE may provide a more selective influence on those cortical circuits previously related to sensory attenuation.

In line with Chambon & Haggard’s (2012) findings of an enhanced experience of control, we expected stronger sense of agency, and therefore more sensory attenuation, when primes and targets were compatible, compared to when they were incompatible. Note that the positive effect of primes on sense of agency would thus coexist with a negative effect on motor performance and on perceived intensity.

## 2 Methods

### 2.1 Participants

We recruited sixteen healthy volunteers (age 22.8 ± 3.2 years (mean ± SD), eight females). All participants were recruited via an online database. They gave written informed consent prior to participation with the right to exit the study at any time. The study was approved by the local ethics committee (University College London, UK). Participants received £10 per hour as reimbursement.

### 2.2 Task

All stimuli were generated and the paradigm was programmed using Presentation® software (Neurobehavioral Systems, www.neurobs.com). The experiment was run in a dark and sound-attenuated room. The task was presented on an LCD screen at a vertical refresh rate of 60 Hz. The screen background colour was white. Participants were seated 75 cm in front of the screen and kept their chin on a chin rest throughout the experiment.

The experiment consisted of a training session, a pre-test, the experimental task and a control task, in this order. In the experimental task, participants performed a temporal two-alternative forced choice task on the loudness of sine wave tones whose onset timing and frequency were determined by participants’ button presses. These button presses were subliminally primed. The training session served to familiarise participants with contingencies between their button presses and the onset timing and frequency of subsequent sine wave tones. In the pre-test, individual loudness discrimination thresholds were determined by means of an adaptive procedure and then used as constant stimulus levels in the experimental task. The control task tested conscious recognition of the primes.

#### 2.2.1 Action priming

Action priming was implemented as follows. Throughout all parts of the experiment, i.e. the training session, the pre-test, the experimental task and the control task, visual targets (black arrow outlines pointing to the right or to the left) were first preceded by visual primes and then by a metacontrast mask. Primes consisted of black arrows pointing either in the same direction as targets or in the opposite direction. In the training session and pre-test, superimposed arrows pointing in both directions served as neutral primes. The metacontrast mask was a black rectangle framing two white superimposed arrows that were pointing in both directions. All visual stimuli were presented at fixation. Primes subtended a visual angle of 2.84° ∗ 1.18° and were presented for one frame (17 ms). Primes were replaced by the fixation cross (font size 1°) for two frames (33 ms), which in turn was followed by the metacontrast mask for seven frames (117 ms), subtending a visual angle of 3.18° ∗ 1.66°. After an SOA of nine frames (150 ms), the target was presented. Targets subtended a visual angle of 5.3° ∗ 1.95° and were presented for seven frames (117 ms).

The experimental factor was the compatibility between prime direction and target direction.

#### 2.2.2 Training session

In the training session (32 trials), participants learnt the contingency between their left or right index finger button presses, as instructed by the direction of the visual targets, and the presentation of a sine wave tone of one of two frequencies (900 Hz and 750 Hz; 100 ms duration) via headphones. In this training session, participants performed two tasks in parallel. On each trial, they pressed a button either with their right or left index finger in response to the direction of the visual target. They were instructed to perform these button presses within one second after target onset and as quickly and accurately as possible. When pressing the wrong button (the button operated by the index finger opposite to the direction of the target) or when pressing the button too late (later than 1200 s after target onset) a red “x” was presented at fixation (font size 1°). As in the pre-test and in the experimental task, these trials were repeated at the end of the regular 32 trials. Index finger button presses were followed by one of two sine wave tones after 50 ms. For half of the participants, left index finger button presses led to the presentation of the low-frequency tone and right index finger button presses to the presentation of the high-frequency tone in the majority of trials of this training session, while the opposite mapping was used for the other half of participants. In the remaining three to five trials of the training session the reverse button – tone frequency mapping was valid (catch trials). The number and distribution of catch trials was randomly determined for each participant. Participants had to count these catch trials and report their number at the end of the training session. This task of counting catch trials was introduced to ensure that participants attended to the button – tone frequency contingencies. Only very few participants miscounted the number of catch trials. All of these were allowed to continue without repeating the training session after ensuring that they gave a correct account of the button – tone frequency mapping.

#### 2.2.3 Pre-test

In the pre-test (six blocks, 32 trials each) index finger button presses in response to the visual targets led to the presentation of a pair of tones of identical frequency, which was now fully determined by the (instructed) choice of buttons. The button – tone frequency mapping that was predominant in the training session was now valid on every single trial, so that participants did not encounter any further reversals of the mapping (no catch trials). The second tone followed the first after a fixed SOA of 1100 ms to keep predictability of the onset timing of the two tones identical. Within each tone pair, one of the two was louder than the other. After the second tone, a question mark was presented in the centre of the screen, prompting participants to indicate whether the first or second tone was the louder by pressing one of two additional buttons with the middle finger of the right hand (these additional buttons were arranged orthogonal to the index finger buttons in order to minimise bias in middle finger button choice by the preceding index finger button press). There was no time limit for the loudness discrimination task. Participants received feedback for this loudness discrimination task after each trial (correct responses were indicated by a green tick, incorrect responses by a red “x”, both at fixation, font size 1°) and after each block (as the average percentage of correct responses in the previous block). In both the training session and the pre-test, only neutral primes (superimposed arrows pointing in both directions) were used.

In this pre-test, stimulus levels were continuously adjusted using a weighted up/down staircase method (3 up, 1 down) (Kaernbach, 1991). There were four different staircases, as sample tones that were either louder or softer than the standard tone (74 dB SPL) were used in separate staircases for each of the two tone frequencies. Initial step size was 6 dB SPL (labelled “delta_firststep_” below). Target direction, order of standard and sample tones and order of the four staircases were pseudo-randomly distributed across each block so that none of these were predictable from any of the others on a given trial. The downward step size was increased during the first two downward slopes of each staircase (3 up, 3 down) under the assumption that initial differences in loudness (corresponding to volume differences of ±6 dB) were easily detectable. The corresponding first reversal points of the staircase were excluded from analysis. Sound volume was calibrated to dB SPL using a sound pressure level meter to arrive at a volume of the standard tone of 74 dB SPL. During the pre-test, loudness of the sample tones was adapted exponentially, approaching 74 dB SPL asymptotically from above and below with improving performance: At any point of the staircase, sound volume of the sample tone was determined by (74 dB SPL ± delta_firststep_ ∗ 1.15^exponent^), with the “±” determining whether the sample was louder or softer than the standard. Exponents started at zero and were varied by the 3 up, 1 down-manipulation (3 up, 3 down for the first two downward slopes), i.e., the exponent of the current staircase was decreased by 1 after a correct response and increased by 3 after an incorrect response. Calibration to dB SPL was checked on each testing day. Playback latency of the sound card was determined to be below 1 ms in preliminary testing.

After the pre-test, the discrimination threshold was determined as the average of all reversals (from decreasing to increasing volume differences and vice versa) from the third reversal onwards, for each of the four staircases separately. Results from this staircase procedure, i.e. threshold values and the number of reversals on which they were based, are reported in Section 3.

#### 2.2.4 Experimental task

**Fig. 1:**
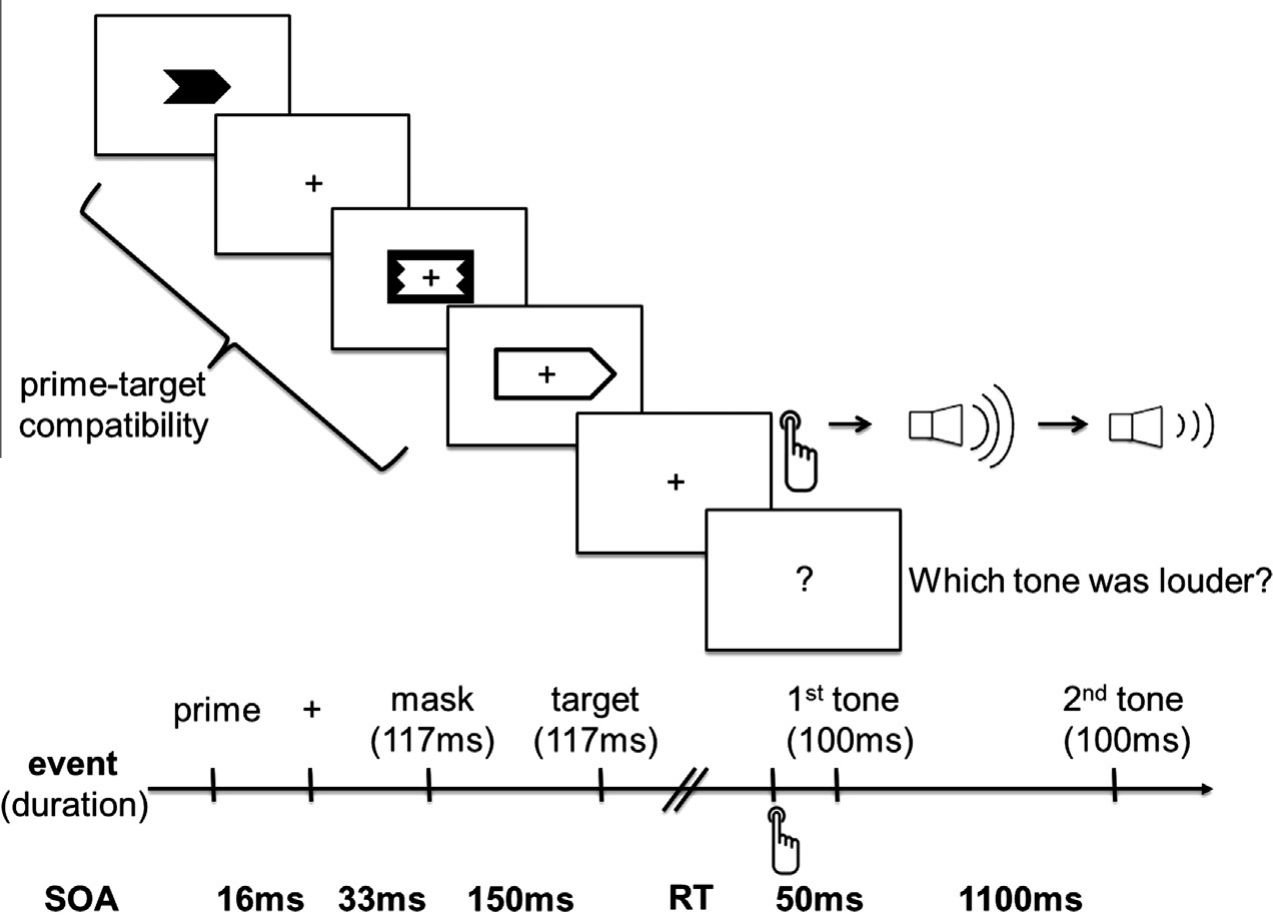
Task design and timing of trial events. The course and timing of events in a trial with a compatible prime–target pair is schematically illustrated. See methods, Section 2.2, for details.

The experimental task (Fig. 1) was identical to the pre-test except for four differences. First, there was no trial feedback for the loudness discrimination task (only the blockwise feedback, i.e., the average percentage of correct trials). Second, and unbeknown to participants, only the second tone was varied in loudness (softer or louder than the first), while the first tone was kept constant at 74 dB SPL. Third, the experimental task was based on the method of constant stimuli, using individual discrimination threshold values from the pre-test for each of the two tone frequencies and the two stimulus levels (softer or louder than the first). And fourth, primes were now either compatible or incompatible with the targets.

The experimental task consisted of six blocks of 32 trials each. Stimulus timing was carefully monitored and sporadic trials with longer prime-mask or mask-target SOAs (due to sporadic asynchrony of the visual output with the vertical refresh rate) were excluded (two trials across all participants and both experiments). Prime direction, target direction and the four threshold values (obtained from the four staircases in the pre-test) were pseudo-randomly distributed across each block, so that none of these were predictable from any of the others on a given trial.

#### 2.2.5 Control task: prime recognition

After the experimental task, existence of the primes was revealed to participants and a forced choice prime recognition task was added (the control task). Visual stimulation was identical to the experimental task, but participants were instructed to ignore the direction of the target arrows as well as the mask and to focus on the direction of the primes only. They were required to indicate whether primes pointed to the right or the left on each trial (by using the respective index finger buttons). There was no time limit for these responses. However, participants had to wait for a tone (875 Hz, 150 ms, presented 600 ms after the onset of the target) before responding. This constraint for reaction times was introduced to keep the influence of subliminal priming on the button choice to a minimum and follows procedures used in previous studies for the same purpose (Chambon and Haggard, 2012, Vorberg et al., 2003, Wenke et al., 2010). There was no feedback for this prime recognition task. The prime recognition task consisted of three blocks, each of 32 trials.

### 2.3 Data analysis

Data on loudness discrimination in the experimental task and on prime recognition in the control task were analysed using signal detection theory measures (Green & Swets, 1966).

Participants whose sensitivity for prime direction in the prime recognition task exceeded a threshold (*d*′ > 0.5) were excluded (three participants in experiment 1 (*d*′ of 3.77, 1.49 and 1.19).

The main behavioural metric of interest relating to the experimental task was the bias to report the first of the two tones as the softer as a measure of sensory attenuation. Sensory attenuation has been known to decay over the course of a few hundred milliseconds (Aliu et al., 2009, Bays et al., 2005). The bias to report the first tone, which was presented 50 ms after the action, as the softer one (relative to the second tone 1150 ms after the action) can therefore reflect sensory attenuation. We used the log likelihood ratio ln(*β*) as a measure of bias, i.e., the natural logarithm of the ratio of the values of the two signal distributions at the decision criterion (Macmillan & Creelman, 2005). In the widely-used (Wickens, 2002) equal-variance Gaussian model of the two distributions, ln(*β*) equals half of the difference of the squared *z*-transforms of the false alarm rate (*F*) and hit rate (*H*):

$$\ln(\beta )=0.5*(z^{2}(F)-z^{2}(H))$$

Systematic differences of ln(*β*) between conditions were not explained by systematic changes in sensitivity (*d*′) as *d*′ was not affected significantly by condition in both experiments (see results).

Effects of prime–target compatibility on reaction times, error rates and ln(*β*) were tested with dependent samples *t*-tests across subjects. A one-tailed *t* test was used where the direction of the effect has previously been established in the previous literature, i.e., when testing for priming effects on reaction time and error rates. For priming effects on our measure of sensory attenuation, a two-tailed *t* test was used. Within-subject effects were tested using independent samples *t*-tests.

## 3 Results

Our main focus was on the bias to report the first of the two tones in the experimental task as the softer. We also measured prime–target compatibility effects on inverse efficiency, a measure that efficiently combines response speed and accuracy (Townsend & Ashby, 1983). We report results relating to bias under “Experimental task”. For completeness, we first report results of the pre-test.

### 3.1 Pre-test

The staircase procedure of the pre-test resulted in four loudness discrimination thresholds for each individual, representing volume levels above and below the volume of the standard tone (74 dB SPL) for each of the two tone frequencies (900 Hz vs. 750 Hz). Loudness discrimination thresholds were not significantly modulated by these two factors (factor 1: volume of the sample tone with respect to the volume of the standard tone [louder, softer], factor 2: tone frequency [900 Hz, 750 Hz]) as revealed by a two-way repeated measures ANOVA. On average, discrimination thresholds were based on 16.3 reversals (averaged across the four staircases and all participants). There were no significant differences in the number of reversals between the four staircases (all *p* > .25). Mean discrimination thresholds for the four staircases were 1.19 dB SPL (750 Hz, staircase approaching 74 dB SPL from below), 1.05 dB SPL (750 Hz, staircase approaching 74 dB SPL from above), 1.02 dB SPL (900 Hz, staircase approaching 74 dB SPL from below) and 1.1 dB SPL (900 Hz, staircase approaching 74 dB SPL from above).

### 3.2 Experimental task

**Fig. 2:**
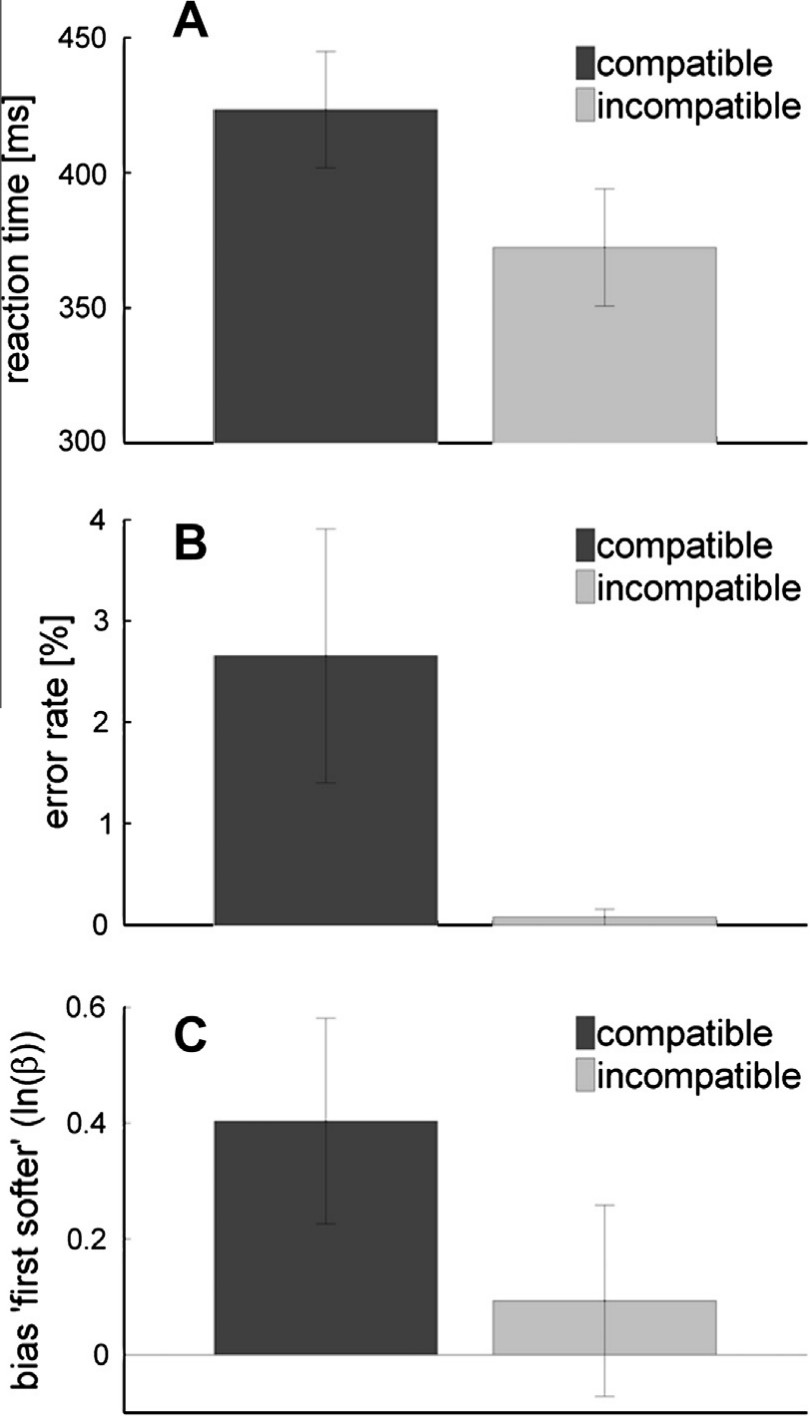
Motor priming effects on motor efficiency and bias in the loudness discrimination task. Prime–target compatibility effects on (A), reaction time (in ms) and (B), error rate (in %) in the speeded reaction time task (responses to the target arrows). (C) Motor priming effects on the bias (ln(*β*)) to report the first of the two tones as the softer one.

As expected, prime–target compatibility significantly modulated efficiency of instructed button presses. Consistent with the literature on negative compatibility effects, compatibly primed responses were significantly less efficient than incompatibly primed responses (*t*_12_ = 6.5, *p* < .001; inverse efficiency (mean ± SD): 433.95 ± 70.17 ms (compatible primes) vs. 372.59 ± 78.13 ms (incompatible primes)). Participants responded significantly more slowly to compatibly primed targets when compared to incompatibly primed targets (*t*_12_ = 8.14, *p* < .001; reaction time (mean ± SD): 423.33 ± 77.64 ms (compatible primes) vs. 372.32 ± 78.23 ms (incompatible primes)) (Fig. 2a) and with a significantly higher error rate (*t*_12_ = 2.05, *p* = 0.032; error rates (mean ± SD): 2.65 ± 4.52% (compatible primes) vs. 0.08 ± 0.28% (incompatible primes)) (Fig. 2b). Eleven (out of thirteen) participants showed a significant negative compatibility effect in a fixed-effect analysis (independent samples *t*-test across trials).

Importantly, prime–target compatibility also significantly modulated bias in our loudness discrimination task. The bias to report the first of the two tones as the softer one was increased in trials in which primes and targets were compatible when compared to incompatibly primed trials (*t*_12_ = 2.34, *p* = .037) (Fig. 2c). Sensitivity, on the other hand, as indexed by *d*′, was not affected significantly by prime–target compatibility (*t*_12_ = 1.49, *p* = .16). Thus, compatibility of primes and targets decreased motor efficiency and, in parallel, increased the bias to report the first of two auditory action consequences as the less intense, i.e., softer.

## 4 Discussion

We demonstrate that the perceived loudness of an auditory consequence of a motor action is attenuated by an experimental manipulation that selectively influences *motor* processing, in the absence of any changes to stimulus predictability. Specifically, we show that perceived loudness and motor efficiency are both decreased in parallel when the action is primed by a compatible, subliminally presented, visual prime–target pair with a long SOA (Eimer, 1999, Lingnau and Vorberg, 2005, Schlaghecken and Eimer, 2000). In previous studies (Chambon and Haggard, 2012, Wenke et al., 2010), this experimental manipulation has been shown to enhance explicit judgements regarding the degree of control over sensory stimuli. However, such explicit judgements may be subject to post hoc interpretative reconstruction. Therefore, they do not provide a direct measure of sense of agency. In contrast, sensory attenuation provides a low-level sensory measure related to sense of agency (Synofzik et al., 2008), with the advantage of a clear link to a strong theoretical model that identifies specific aspects of action processing (i.e., the feed-forward predictor and the comparator stage). Several other studies have appreciated the value of implicit, perceptual measures of the sense of agency (Shergill et al., 2005), but we believe ours may be the first such study to do so while avoiding any confounding effects related to action-stimulus contingency.

### 4.1 Deconfounding experimental studies of sense of agency

Our study was partly motivated by concerns about confounds in previous studies on sensory attenuation. Sensory attenuation is, by definition, action-specific (Bays et al., 2005, Chapman et al., 1987) and studies on sensory attenuation should be able to unambiguously attribute observed changes in perception to motor actions. Ideally, all parameters of an action-stimulus sequence should remain constant across experimental and control conditions to avoid confounding those effects that are due to the manipulation of interest – e.g. due to manipulations of experienced stimulus causation – and those that arise from any changes to stimulus presentation with respect to the action.

Despite such potential pitfalls, most previous studies on sensory attenuation are based on study designs that manipulate the contingency between an action and a stimulus and may therefore lead to different levels of stimulus predictability. Given the ubiquitous perceptual effects of predictability, these confounds make judgements of stimulus intensity problematic. For example, some previous studies on sensory attenuation have compared stimuli that are contingent on an action (in “motor-to-effect” conditions) to identical stimuli that are not preceded by a motor command of the observer (in “effect only” conditions) (e.g. Weiss et al., 2011). This design could confound authorship over action consequences with motor processing. Other studies have tried to avoid this confound by manipulating the “when” or “what” of action consequences, i.e., the time lag between the action and its consequence (e.g. Bäss et al., 2008) or the mapping of various actions onto different consequences (e.g. Cardoso-Leite et al., 2010). Such designs successfully control for predictability of stimulus *occurrence*, but still need to control for predictability of stimulus onset timing, or stimulus identity (for a review of these confounds in previous studies, see Hughes et al., 2013).

A recent EEG study used subliminal priming of sensory action consequences (rather than priming of the motor action itself, as in our study) to demonstrate a neurophysiological correlate of sensory attenuation (of visually evoked responses) (Gentsch & Schütz-Bosbach, 2011). The concept of modulating the experience of control over stimuli by (subliminal or conscious) priming of these action consequences prior to the action stems from studies on explicit agency judgements (Aarts et al., 2005, Linser and Goschke, 2007, Wegner and Wheatley, 1999). However, priming of sensory action consequences (“effect priming” rather than motor priming) might by-pass specifically action-mediated effects on perception and alter sensory processing directly, i.e., via stimulus–stimulus associations, which are potentially independent of the action. Studies that are based on priming of sensory action consequences (rather than the motor action itself) cannot unambiguously attribute observed sensory effects to the preceding action. This reservation remains even if no effect of the sensory priming is observed in control conditions that lack the action (since these control conditions raise similar issues as studies that compare “motor-to-effect” conditions and “effect only” conditions, as described above).

In contrast, our study design dissociates the primary target of the experimental manipulation – the action – from the outcome measure of interest – perceived loudness – and thus provides strong support for the proposal that perceived loudness is indeed modulated *through the action*, as our measures of sensory attenuation and motor performance co-varied with prime–target compatibility.

### 4.2 Motor processing and negative compatibility priming

We hypothesised that sensory attenuation would be stronger when actions were compatibly primed. This hypothesis was based on the finding that the experience of control is enhanced when primes and targets are compatible (Chambon & Haggard, 2012) and on the proposed association of sensory attenuation and the sense of agency (Shergill et al., 2005). Direct support for this specific direction of the priming effect on sensory attenuation comes from studies on motor priming.

A model of self-inhibitory neural circuits has been proposed to explain negative compatibility effects on motor performance (Eimer & Schlaghecken, 2003), based on recordings of the lateralized readiness potential (LRP) in conjunction with behavioural measures of motor efficiency (Eimer & Schlaghecken, 1998). According to this model, an initial facilitation of the correct motor response automatically turns into inhibition of the correct response when primes and targets are further separated in time (but see Jaśkowski, 2008). This prime-induced, automatic, inhibitory effect has been considered to be dissociable from the effects of resolving the subsequent response conflict induced by prime–target compatibility (D’Ostilio et al., 2012).

In our study, only trials that showed correct responses to targets were taken into account. These were trials in which the conflict between automatic motor inhibition and target instruction was successfully resolved and motor inhibition was overcome. Previous studies have demonstrated that physiological signals of motor processing are enhanced when response conflict induced by prime–target compatibility is resolved in favour of the correct response. More specifically, an increase in blood-oxygen-level-dependent signal in the supplementary motor area has been shown (Boy, Evans, et al., 2010) as well as a larger amplitude of the lateralized readiness potential at the time of the response (Figs. 2 and 4 in Eimer and Schlaghecken, 1998, Eimer and Schlaghecken, 2003, respectively) in compatible vs. incompatible trials. This suggests an enhanced neural representation of the chosen action in compatible vs. incompatible trials when auto-inhibition is overcome, and, consecutively, may imply higher accuracy (Blakemore, Wolpert, & Frith, 2000) or precision (Brown et al., 2013) of sensory predictions. In line with this, we find stronger sensory attenuation when actions are compatibly primed.

### 4.3 Bias and sensitivity in sensory attenuation

We used bias in a discrimination task as a measure of sensory attenuation, similar to previous studies that reported shifts in the point of subjective equality (Desantis et al., 2012, Haggard and Whitford, 2004, Weiss et al., 2011). Early studies on sensory attenuation reported detection rates and magnitude estimation as outcome measures (Chapman et al., 1987), which can, in principle, reflect either sensitivity or bias or both (Macmillan & Creelman, 2005). Indeed, a prominent example of sensory attenuation, the force-matching illusion (Shergill, Bays, Frith, & Wolpert, 2003), is a shift of both the point of subjective equality and the slope of the psychometric function.

From a theoretical perspective, the comparator model predicts signal cancellation, not a change in the sensory gain function (but see Brown et al., 2013). The effects of signal cancellation on the two main signal detection theory measures, sensitivity and bias, depend on the choice of task design. In a detection paradigm, both signal cancellation and gain reduction predict a decrease in sensitivity. Accordingly, a previous study that was based on a detection paradigm found effects of response – stimulus congruency (with respect to a learnt mapping) on sensitivity (Cardoso-Leite et al., 2010). In discrimination paradigms, however, mere cancellation of a sensory signal leads to a perceptual bias, whereas a change of sensory gain affects both signal amplitude *and* variance and, thereby, discriminability from the reference stimulus, i.e., discrimination sensitivity.

Experiments that require high predictability of the signal for conceptual reasons, such as studies on sensory attenuation, may profit from a design based on signal discrimination, as a discrimination task allows to present the same signal on every trial and to vary the reference stimulus only. Furthermore, absence of an effect on sensitivity in signal discrimination tasks, as in our study, speaks against any major, potentially confounding effects on attention. Attentional effects would typically affect the signal-to-noise ratio, i.e., the sensory gain function (Hillyard, Vogel, & Luck, 1998), resulting in altered sensitivity.

In principle, phenomena interpreted as sensory attenuation might occur at an early sensory level as well as at higher, decision-related stages. As outlined above, previous studies interpret both sensitivity and bias effects as sensory attenuation. Studies that show a decrease of early electrophysiological components of sensory processing typically suffer from those potential confounds, e.g. by stimulus predictability, that our experiment aimed to address (e.g. Gentsch & Schütz-Bosbach, 2011). Taken together, the previous literature on sensory attenuation cannot conclusively attribute sensory attenuation phenomena to purely perceptual or pure decision processes.

Our study was not designed to dissociate between perceptual and decision-related components of sensory attenuation. We focussed more on the motor locus of the signals causing sensory attenuation than on the sensory (or any other) locus where the attenuating effect occurred. However, our results make a purely decisional effect unlikely, since response mappings in the speeded reaction time task and in the loudness discrimination task were orthogonal (indeed, buttons for the two tasks were arranged orthogonal to each other).

### 4.4 Priming and unconscious processing

Our results are also relevant to the wider questions of how unconscious stimuli can modulate cognition (Kouider & Dehaene, 2007). Previous studies have demonstrated that subliminal priming can influence perceptual, lexical, semantic and motor processing (Dehaene et al., 1998, Kouider and Dehaene, 2007). Wenke et al. (2010) and Chambon and Haggard (2012) further showed that unconscious perception can affect conscious experience of control over the consequence of an action. The authors argued that such action priming effects were mediated by signals arising from action preparation, i.e., from within the motor system. Here, we demonstrate that subliminal priming effects extend beyond motor processing to the conscious perception of the consequence of an action.

Would supraliminal primes produce similar effects to those we have observed with subliminal primes? Previous studies with the NCE disagreed regarding the relation between prime visibility and priming effects on motor performance (Eimer and Schlaghecken, 2002, Schlaghecken et al., 2008). Here, we excluded three participants based on relatively high sensitivity in the prime recognition task. Our rationale for excluding these participants was that Wenke et al. reported very different results of motor priming on the experience of control depending on prime visibility (2010). In line with this observation, we found a trend towards a significant difference in the priming effect on our measure of sensory attenuation between participants who were excluded and the rest (independent samples *t*-test, *t*_14_ = 2.11, *p* = .053; mean shift in bias from compatible to incompatible trials, ±SD: −0.32 ± 0.41 (excluded participants) and 0.31 ± 0.48 (included participants)). This offers some circumstantial evidence that consciously seeing the primes reverses their effect on perceived outcome intensity. Whereas subliminal primes produce a negative compatibility effect on both motor performance and sensory attenuation, supraliminal primes have, if anything, the opposite effect. This may indicate that prime awareness is a predictor of (the direction of) priming effects on sensory attenuation. However, this is just circumstantial evidence and should be treated with caution: stronger evidence would come from an experiment designed to address the issue of prime visibility. In the meantime, it is possible that the internal, motoric sense of agency studied here operates in a form of competitive inhibition with other, external attributions of consequent stimuli. A supraliminal prime might provide an alternative potential cause to which the consequent tone could be attributed, and this would then weaken the motoric sense of agency.

In summary, based on a well-controlled, theoretically pure design, our study demonstrates that sensory attenuation of action consequences is driven by top-down modulation of perception *specifically via motor processing*. Conversely, our results show that effects of subliminal motor priming extend beyond motor execution to conscious perception of sensory action consequences. Our approach provides the basis for future studies that focus on behaviourally relevant neural mechanisms underlying sensory attenuation.
